# Regulated Mesenchymal Stem Cells Mediated Colon Cancer Therapy Assessed by Reporter Gene Based Optical Imaging

**DOI:** 10.3390/ijms19041002

**Published:** 2018-03-27

**Authors:** Senthilkumar Kalimuthu, Liya Zhu, Ji Min Oh, Ho Won Lee, Prakash Gangadaran, Ramya Lakshmi Rajendran, Se Hwan Baek, Yong Hyun Jeon, Shin Young Jeong, Sang-Woo Lee, Jaetae Lee, Byeong-Cheol Ahn

**Affiliations:** Department of Nuclear Medicine, School of Medicine, Kyungpook National University, Kyungpook National University Hospital, Daegu 41944, Republic of Korea

**Keywords:** herpes simplex virus thymidine kinase (HSV1-sr39TK), mesenchymal stem cells (MSCs), optical imaging, colon cancer, suicide gene therapy

## Abstract

Colorectal cancer is the most common cancer in both men and women and the second most common cause of cancer-related deaths. Suicide gene-based therapy with suicide gene-transduced mesenchymal stem cells (MSCs) is a promising therapeutic strategy. A tetracycline-controlled Tet-On inducible system used to regulate gene expression may be a useful tool for gene-based therapies. The aim of this study was to develop therapeutic MSCs with a suicide gene that is induced by an artificial stimulus, to validate therapeutic gene expression, and to monitor the MSC therapy for colon cancer using optical molecular imaging. For our study, we designed the Tet-On system using a retroviral vector and developed a response plasmid RetroX-TRE (tetracycline response element) expressing a mutant form of herpes simplex virus thymidine kinase (HSV1-sr39TK) with dual reporters (eGFP-Fluc2). Bone marrow-derived MSCs were transduced using a RetroX-Tet3G (Clontech, CA, USA) regulatory plasmid and RetroX-TRE-HSV1-sr39TK-eGFP-IRES-Fluc2, for a system with a Tet-On (MSC-Tet-TK/Fluc2 or MSC-Tet-TK) or without a Tet-On (MSC-TK/Fluc2 or MSC-TK) function. Suicide gene engineered MSCs were co-cultured with colon cancer cells (CT26/Rluc) in the presence of the prodrug ganciclovir (GCV) after stimulation with or without doxycycline (DOX). Treatment efficiency was monitored by assessing Rluc (CT26/Rluc) and Fluc (MSC-Tet-TK and MSC-TK) activity using optical imaging. The bystander effect of therapeutic MSCs was confirmed in CT26/Rluc cells after GCV treatment. Rluc activity in CT26/Rluc cells decreased significantly with GCV treatment of DOX(+) cells (*p* < 0.05 and 0.01) whereas no significant changes were observed in DOX(−) cells. In addition, Fluc activity in also decreased significantly with DOX(+) MSC-Tet-TK cells, but no signal was observed in DOX(−) cells. In addition, an MSC-TK bystander effect was also confirmed. We assessed therapy with this system in a colon cancer xenograft model (CT26/Rluc). We successfully transduced cells and developed a Tet-On system with the suicide gene HSV1-sr39TK. Our results confirmed the therapeutic efficiency of a suicide gene with the Tet-On system for colon cancer. In addition, our results provide an innovative therapeutic approach using the Tet-On system to eradicate tumors by administration of MSC-Tet-TK cells with DOX and GCV.

## 1. Introduction

Colorectal cancer (CRC) is the third leading cause of cancer death in men and women in the United States and the second most common cause of cancer-related deaths in both men and women world-wide [1]. With regular screening, colon cancer can be detected early, when treatment is most effective. When detected and treated at a later stage, recurrence of the cancer is more common. Therapeutic strategies are available for treatment of CRC; however, the five-year survival rate is still only about 65% (Available online: http://seer.cancer.gov/statfacts/html/colorect.html). Therefore, new therapeutic strategies are urgently needed to improve the prognosis of CRC. In this regard, mesenchymal stem cells (MSCs) carrying therapeutic genes might be one therapeutic option. 

MSCs are multipotent cells that preferentially reside in perivascular niches in all human tissues and organs, including adipose tissue, bone marrow, heart and lung tissue, and neonatal tissues including amniotic membranes, umbilical cords, and placentas [2,3,4,5]. The application of MSCs to drug therapy extends beyond tumor and immune suppression, and because of their tumor-tropic and migratory properties, MSCs have been engineered to express pro-apoptotic, anti-proliferative, and anti-angiogenic factors for localized and systemic treatment of diseases [6,7]. As such, MSCs have become important drug delivery candidates, generating optimism that therapies with greater efficacy can be developed. Of particular note, MSCs have been used in gene therapy [8,9], and experimental studies are being conducted to determine their therapeutic potential in cardiovascular and neurodegenerative disorders [10,11]. One of the main advantages of using MSCs as a drug delivery system is their ability to self-renew, allowing them to act as “self-maintaining drug-delivery vehicles” [12]. Studies have been performed on the delivery of therapeutic genes by MSCs that capitalize on MSC’s self-proliferation and differentiation capacities and that have employed MSCs transduced with therapeutic genes such as tumor necrosis factor (TNF)-related apoptosis-inducing ligand (TRAIL), a trans-membrane protein that causes selective apoptosis in tumor cells [13,14]. 

Gene-directed enzyme prodrug therapy (GDEPT), also known as suicide gene therapy, takes advantage of the properties of MSCs and, combined with a prodrug, offers additional promise as a therapy [15]. This combined system relies on a vector to deliver a transgene and prodrug to the target (i.e., tumor) cell. The suicide gene is transcribed and translated into an active enzyme in the MSCs, which in turn converts the prodrug to its cytotoxic form leading to the death of the MSCs, as well as to the death of nearby cancer cells through a mechanism called the bystander effect, eventually resulting in tumor regression [16]. The most commonly used suicide gene-based therapy incorporates herpes simplex virus type 1-thymidine kinase (HSV1-TK) and the drug ganciclovir (GCV), which is converted into the cytotoxic GCV triphosphate [17].

In order to be of use in patients, therapeutic gene-based systems with enhanced safety are required. The expression of a transduced therapeutic gene needs to be controlled to prevent adverse side effects caused by inappropriate expression of the gene. A tetracycline-inducible system (Tet-system) has been used to control long-term transgene expression in small animal models [18]. This inducible system is regulated by the presence or absence of doxycycline (DOX) (Tet-On or Tet-Off systems). The suicide gene HSV1-sr39TK combined with a Tet-system may therefore be a useful tool for treating cancer with reduced side effects that are commonly related to gene-based therapy. In the current study, MSCs with DOX-inducible HSV1-sr39TK were created and their bystander cytotoxic potential was assessed in colon cancer cells, both in an in vitro and in vivo model, using an optical molecular imaging system.

## 2. Results

### 2.1. Characterization of MSC-Tet-TK and MSC-TK

**Figure 1:**
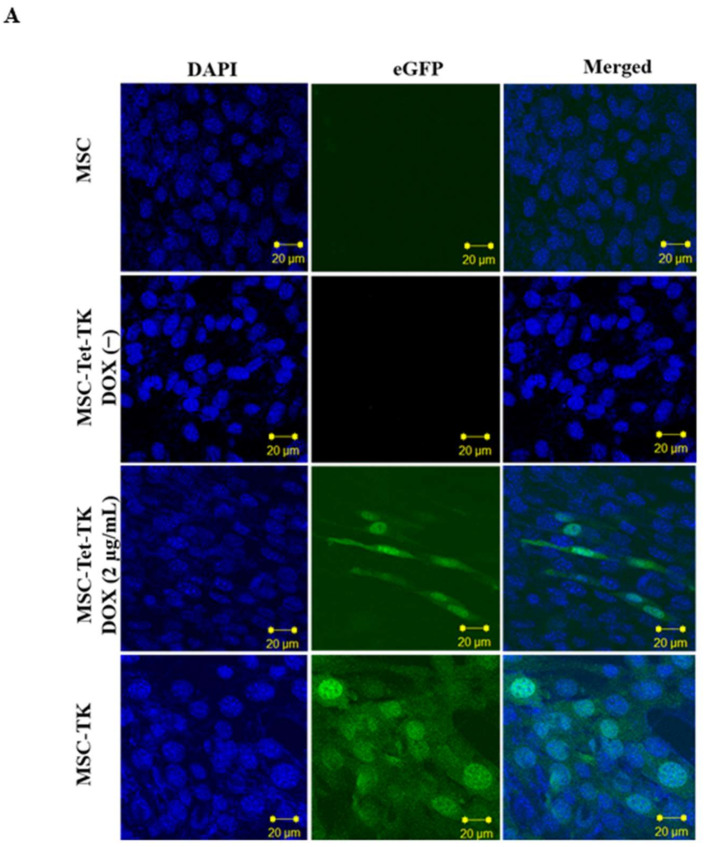

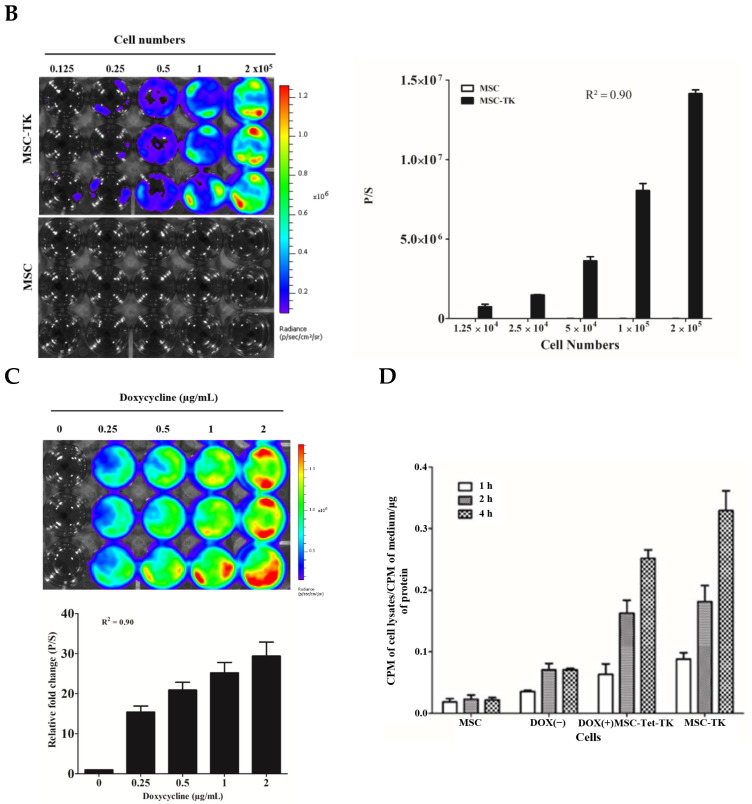
Transduction of mesenchymal stem cells (MSCs) with triple fusion (TF) reporter genes. (**A**) Enhanced green fluorescent protein (eGFP) confocal microscopy images of MSC-TK, MSC-Tet-TK plus DOX (2 µg/mL, 24 h) or doxycycline(−) (DOX(−)), and parental MSC cells. (**B**) Bioluminescent imaging (BLI) of Fluc activity in transduced MSC-TK cells at different cell densities. (**C**) Fluc activity of transduced MSCs (MSC-Tet-TK) after DOX treatment for 24 h assessed by bioluminescent imaging (BLI) and quantitation of this Fluc activity. (**D**) ^3^H penciclovir (PCV) uptake assay. ^3^H-Penciclovir (PCV) uptake of parental MSCs, DOX(−) and DOX(+) MSC-Tet-TK, and MSC-TK cells for 1, 2, and 4 h. p/s, photons/second.

The transduced MSC-Tet-TK and MSC-TK cells were created by retroviral transfection, and eGFP-positive cells were sorted by FACS Aria III. Cells with an eGFP concentration of more than 50% were used for further study. The expression of MSC-TK cell eGFP and MSC-Tet-TK cell eGFP after induction with DOX (2 µg/mL) for 24 h (Figure 1A) was confirmed by confocal microscopy images. The morphology of MSC-Tet-TK cells are heterogeneous and not uniform, because MSCs are a heterogeneous cell population. MSC-Tet-TK cells with eGFP positive revealed more rod-like morphology than others, which might be related to the genetic alteration. Some of the MSC-TK cells revealed higher eGFP signals due to higher eGFP expression, because protein synthesis can vary with individual cells, withdifferent copy numbers of the transduced gene. The Fluc activity of MSC-TK cells increased with increasing cell numbers (Figure 1B, *R*^2^ = 0.90). The BLI signal in MSC-Tet-TK cells increased 15-, 21-, 25-, and 29-fold with increasing concentrations of DOX (0.25, 0.5, 1, and 2 µg/mL), whereas no signal was observed in DOX(−) cells (Figure 1C). Furthermore, we found that Fluc activity increased 17-, 19-, 20-, 20-, 15-, and 7-fold with increasing DOX treatment (0.5 to 4 µg/mL) for 48 h and then decreased at 8 and 16 µg/mL DOX (Figure S1). Therefore, in this study, we elected to use 2 µg/mL DOX for transgene activation.

### 2.2. ^3^H-PCV Uptake Assay

In order to confirm the functional activity of transduced cells, we performed a ^3^H-PCV uptake assay. The suicide gene HSV1-sr39TK converts PCV into penciclovir monophosphate, and, subsequently, cellular monophosphate kinases and diphosphate kinases convert penciclovir monophosphate to the triphosphate form. The accumulation of penciclovir inside the cells indirectly indicates the functional efficiency of the HSV1-sr39TK enzyme expressed in cells. MSC-TK and DOX(+) MSC-Tet-TK cells showed higher ^3^H-PCV uptake compared to naive MSCs and DOX(−) MSC-Tet-TK cells (Figure 1D).

### 2.3. Characterization of CT26/Rluc Cells

The Rluc activities of stably transduced CT26/Rluc and parent CT26 cells were determined by BLI. CT26/Rluc activity increased with increasing cell number (Figure S2A, *R*^2^ = 0.91). mCherry expression was also confirmed by fluorescence microscopy (Figure S2B).

### 2.4. Cell Viability of MSCs after Treatment with DOX and GCV

To confirm drug (GCV and DOX) effects in naive MSCs, we analyzed cell viability. We found that GCV did not affect naive MSC viability (Figure S3A) after 48 h of treatment. Since we used a DOX-inducible gene system for gene activation, we also assessed the effect of DOX on the viability of naïve MSCs. We found that MSC cell viability was reduced significantly at 8 and 16 µg/mL DOX (*p* < 0.05) (Figure S3B).

### 2.5. Fluc Activity of Suicide Gene-Transduced MSCs after Treatment with GCV

**Figure 2:**
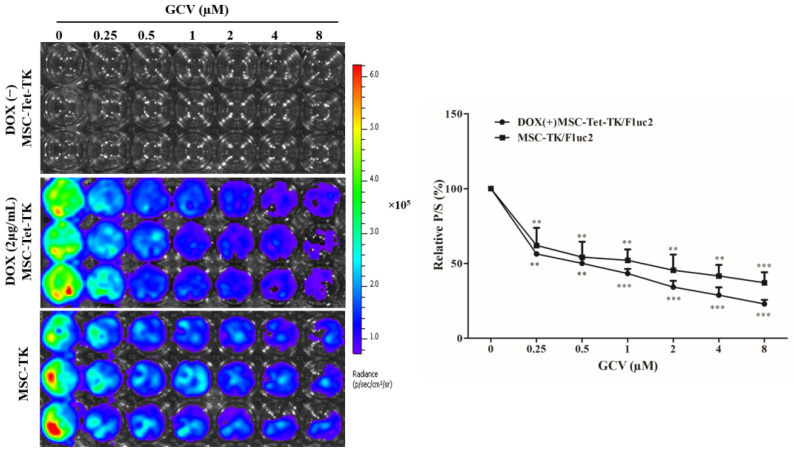
Fluc activity of MSC-Tet-TK and MSC-TK cells after ganciclovir (GCV) treatment for 48 h. Fluc activity was measured by bioluminescent imaging (BLI), and the quantitation for MSC-Tet-TK and MSC-TK cells is shown in the right-hand panel. Values obtained from three experiments are expressed as the mean ± standard deviation (SD), ** *p* < 0.01, *** *p* < 0.001 (by Student’s *t*-test). p/s, photons/second.

The relative Fluc activity of MSC-Tet-TK cells decreased 56, 50, 43, 34, 28, and 22% in DOX(+) MSC-Tet-TK cells treated with increasing concentrations of GCV (0.25, 0.5, 1, 2, 4, or 8 µM, respectively). In contrast, the DOX(−) group did not show any detectable Fluc signal. In addition, the relative Fluc activity of MSC-TK cells also decreased 62, 54, 52, 45, 41, and 37% with increasing concentrations of GCV (Figure 2). Therefore, in this study, we successfully developed MSCs with a Tet-On system (MSC-Tet-TK), and confirmed the induced expression of Fluc in the presence of DOX, as well as the cytotoxic effect of GCV.

### 2.6. Bystander Effects on Colon Cancer Cells with Suicide Gene Expressed by Engineered MSCs

**Figure 3:**
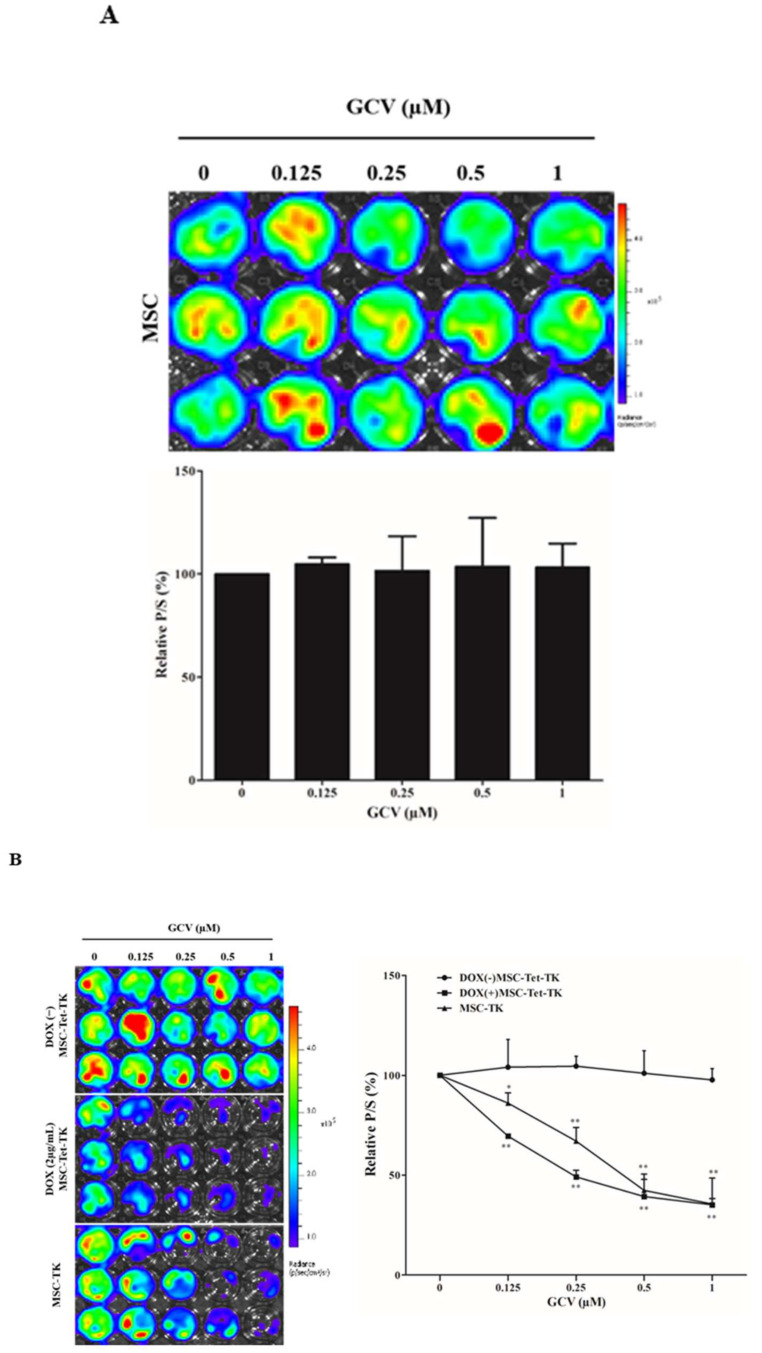
Bystander effect of MSC-Tet-TK and MSC-TK cells. (**A**) Rluc activity in co-cultures (1:1) of naive MSCs and CT26/Rluc cells treated with the indicated concentrations of GCV for 48 h. (**B**) BLI images of the Rluc activity and quantitation data of CT26/Rluc in co-cultures (1:1) of MSC-TK or MSC-Tet-TK cells in the absence or presence of doxycycline (DOX(−) and DOX 2 μg/mL, respectively). Three experiments are expressed as the mean ± standard deviation (SD), * *p* < 0.05, ** *p* < 0.01, *** *p* < 0.001 (by Student’s *t*-test). p/s, photons/second.

The therapeutic effect of MSC-Tet-TK and MSC-TK on colon cancer cells was analyzed. To assess this, we initially co-cultured naïve MSCs with CT26/Rluc cells and treated them with GCV for 48 h to assess the effect of GCV on naive MSCs. The Rluc activity was not changed by GCV treatment, confirming that GCV has no effect on naive MSCs (Figure 3A). Further, to evaluate the bystander effect, we co-cultured either MSC-Tet-TK or MSC-TK cells separately with CT26/Rluc cells at a 1:1 ratio, and increasing concentrations of GCV were administered (0.125 to 1 µM), with or without prior DOX induction. The relative Rluc activity of CT26/Rluc cells significantly declined 69, 49, 39, and 35% (*p* < 0.01) with increasing concentrations of GCV (Figure 3B) in DOX(+) MSC-Tet-TK cells co-cultured with CT26/Rluc cells. However, the relative Rluc activity of CT26/Ruc cells did not decrease significantly (104, 104, 101, and 98%) with increasing GCV concentrations (Figure 3B) in DOX(−) MSC-Tet-TK cells co-cultured with CT26/Rluc cells. In addition, the relative Fluc activity of MSC-Tet-TK cells significantly decreased 61, 54, 48, and 46% with GCV (0.125 to 1 µM respectively), demonstrating the function of the Tet-On HSV1-sr39TK/GCV suicide system in DOX(+) cells. In contrast, there was no Fluc signal observed in the DOX(−) MSC-Tet-TK cells (Figure S4). In addition, the therapeutic effect of MSC-TK cells was also monitored in CT26/Rluc cells, and the Rluc activity was found to be decreased by 86, 67, 42, and 35%, respectively (*p* < 0.05, 0.01), after 48 h of GCV treatment (Figure 3B). The relative Fluc activity of MSC-TK cells also significantly decreased 98, 76, 68, and 43% after 48 h of GCV treatment (Figure S4). Therefore, these studies confirmed the bystander effect of MSCs expressing the suicide gene (MSC-Tet-TK or MSC-TK) in an in vitro colon cancer cell line.

### 2.7. Effects of Suicide Gene-Transduced MSCs on Colon Cancer in a Mouse Model

**Figure 4:**
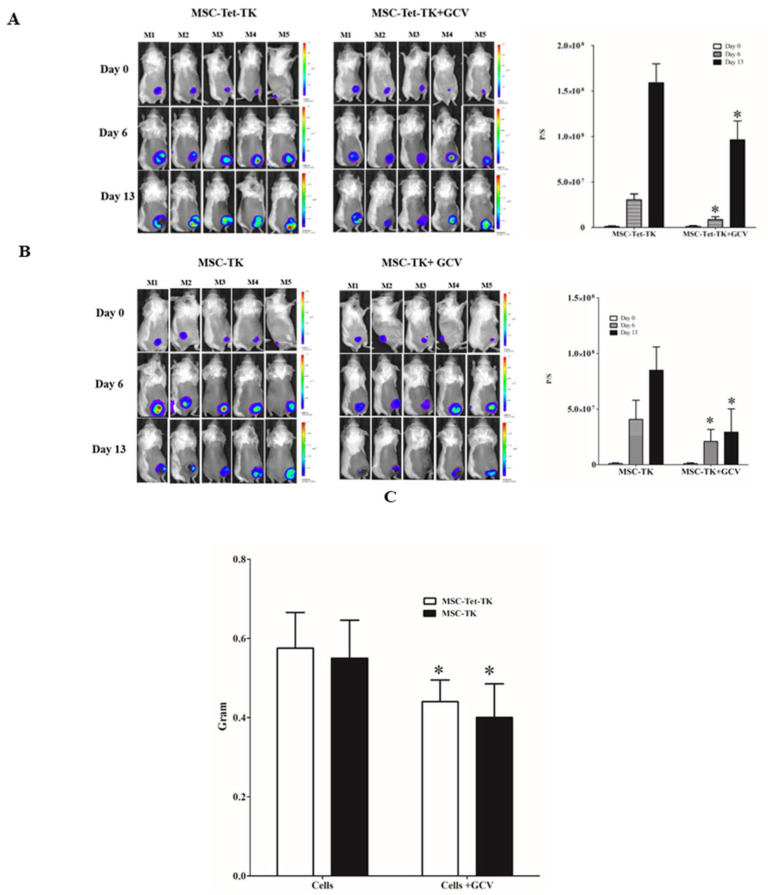
In vivo therapeutic effect of MSC-Tet-TK and MSC-TK cells on inhibiting colon tumor growth. (**A**) Renilla luciferase (Rluc) imaging of colon cancer cells (CT26/Rluc) in mice treated with either MSC-TK or MSC-Tet-TK cells with or without concurrent GCV treatment. BLI images were taken on days 0, 6, and 13 in five individual mice; (**B**) Quantitative analysis of the data shown in (**A**). (**C**) Tumor weights assessed at study end. Bioluminescence activity is shown in photons/second (p/s). * *p* < 0.05 compared separately to MSC-Tet-TK (GCV−) and MSC-TK (GCV−).

To determine the effect of suicide gene-transplanted MSCs on colon cancer growth, Rluc imaging was used to monitor tumor progression. After suicide gene-transduced MSC transplantation and GCV treatment, we found, by monitoring CT26/Rluc activity using BLI, that tumor growth was inhibited in the GCV+DOX-treated MSC-Tet-TK group (Figure 4A) compared to that in the DOX-only treatment group. The MSC-TK+GCV-treated group also exhibited significantly decreased Rluc activity compared with that of the MSC-TK group (Figure 4B). We also monitored the survival of MSC-Tet-TK cells on day 1 by measuring Fluc activity. The Fluc activity on day 1 was very high in mice injected with either cell type (MSC-Tet-TK and MSC-TK), but declined after administration of GCV compared to mice injected with the cells alone (Figure S6A,B). Tumor weights were significantly (*p* < 0.05) reduced in the MSC-Tet-TK+DOX+GCV-treated group compared with those in the MSC-Tet-TK+DOX group (Figure 4C). The body weight of the treatment group was lower than untreated group, but it was not statistically significant (data not shown).

### 2.8. In Vivo Tumor Apoptosis Analysis

**Figure 5:**
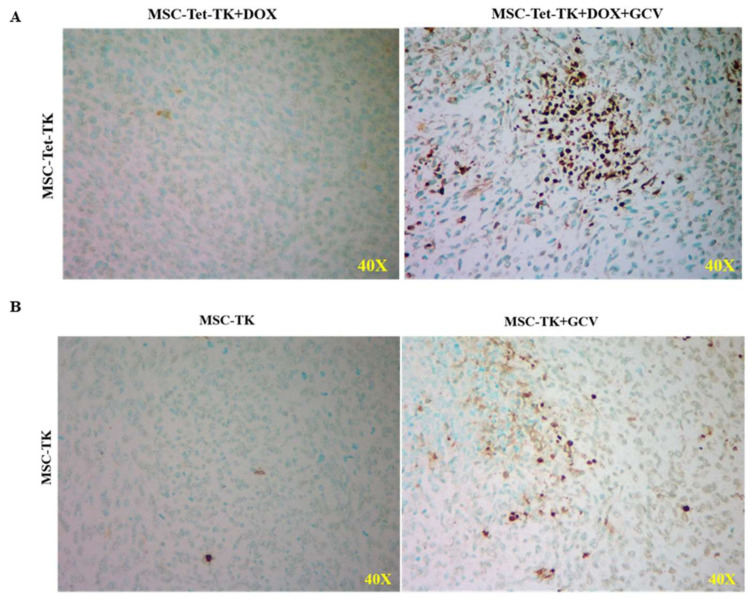
In vivo therapeutic effect of the MSC-Tet-TK and MSC-TK cells to induce colon tumor apoptosis in the presence of GCV. (**A**) Terminal deoxynucleotidyl transferase (TdT) dUTP Nick-End Labeling (TUNEL) assay for tumors taken from mice treated with MSC-Tet-TK DOX cells in the presence and absence of GCV. (**B**) TUNEL assay for tumors taken from mice treated with MSC-TK cells in the presence and absence of GCV. Apoptosis of cells was detected in the ganciclovir-treated group using the ApopTag peroxidase in situ apoptosis detection method.

To further confirm the antitumor effect of transduced MSC-mediated apoptosis in vivo, we utilized a tumor apoptosis assay. The ApopTag Peroxidase In Situ Apoptosis Detection Kit confirmed in vivo tumor apoptosis in the transduced MSC and GCV treatment groups; the effect was enhanced by the application of GCV. This result was confirmed by TUNEL staining. Therefore, our results suggest that suicide gene-transduced MSCs with (MSC-Tet-TK) or without the Tet-On system (MSC-TK) inhibit tumor growth by inducing cell apoptosis in vivo (Figure 5A,B).

## 3. Discussion

In this current study, we successfully established an “on” or “off” switch for the suicide gene HSV1-sr39TK MSCs also co-expressing a Fluc2 reporter gene, and we assessed the therapeutic effect of this system, both with and without the Tet-On function, in a pre-established tumor using optical imaging. For this study, we used a retroviral vector to stably transfect cells and then selected the transduced MSCs for further use. The FACS-sorted immortalized MSCs expressing HSV1-sr39TK exhibited in vitro susceptibility to GCV [19]. In the present study, we selected the transduced MSCs (MSC-Tet-TK and MSC-TK) after induction with or without DOX and confirmed the cell-killing effect of GCV following DOX induction. We also showed that suicide gene-transduced MSCs played an effective antitumor role in a human colon cancer xenograft model by inducing cell apoptosis in xenograft tumor cells. The colon cancer cells used in this study had previously been transduced with mCherry-Rluc using lentiviral particles. The MSCs were introduced into the tumor by direct injection. The MSCs and CT26 tumor cells could each easily be monitored by measuring their respective Fluc and Rluc activities by bioluminescent imaging (BLI) using the substrates d-Luciferin and coelenterazine, respectively [20,21,22,23].

The clinical safety of traditional constitutively overexpressed therapeutic genes is often compromised by side effects related to the failure to control the timing and level of gene expression, which are critical for the functions of genes [24,25]. The Tet system can be used to induce gene expression and transcription, and to reversibly turn on or off expression in the presence of an inducing factor such as tetracycline or doxycycline (DOX) [26]. In this study, we used the “Tet-On 3G” protein, which is considerably more sensitive to DOX than the native Tet-On system and provides for much higher expression of the HSV1-sr39TK suicide gene [27]. We assessed the effect of the pro-drug GCV in the genetically transduced MSCs (either with or without the Tet-On system, namely MSC-Tet-TK and MSC-TK cells, respectively), using BLI. The results of our study showed that these suicide gene-expressing MSCs responded to the prodrug in a dose dependent manner (Figure 2).

Next, we studied the consequence of prodrug conversion to its cytotoxic form inside the suicide gene-transduced MSCs by assessing the ability of these MSCs to kill neighboring cancer cells (the so-called bystander effect). The results showed that MSCs either with (MSC-Tet-TK) or without (MSC-TK) the DOX system significantly induced cell death in co-cultures with colon cancer cells (CT26/Rluc) following treatment with increasing concentrations of GCV, as evidenced by decreases in Rluc imaging and relative Rluc activity in the co-cultured colon cancer cells (Figure 3B). Importantly, GCV did not have any effect on Rluc activity in co-cultures of colon cancer cells (CT26/Rluc) with naive MSC (Figure 3A). MSCs expressing the suicide gene HSV1-TK that are sensitive to GCV can then facilitate the permeation of phosphorylated GCV to neighboring cells that do not express the suicide gene, a phenomenon known as the bystander effect that has been observed in U-87 brain cancer cells in the presence of GCV [28]. Gap junctions may exist between MSCs and the colon cancer cells used here because the bystander effect induced by GCV triphosphate has been shown to be dependent on active transport via gap junction intercellular molecules [29]. This is also supported by the findings of another study by Matuskova et al., who demonstrated the formation of gap junctions between MSCs and cancer cells after co-culture [30]. Our data, therefore, confirmed the bystander effect in co-cultures of MSCs with colon cancer cells in vitro.

One advantage of our system is that therapeutic gene expression was linked with expression of a reporter gene Fluc2, meaning that expression of the therapeutic gene could be monitored simply by measuring Fluc expression. Therefore, the combination of the inducible Tet system and the fluorescent reporter gene allow for very easy control of the expression of the therapeutic gene, and this might be helpful in reducing side effects related to the expression of the therapeutic gene. In addition, the molecular imaging approach used in this study is ideally suited to evaluate in vivo tumor progression and regression in the evaluation of new cancer therapies [31,32,33,34].

Kucerova et al. successively applied retrovirus-transfected with cytosine deaminase (CD) into human adipose tissue MSCs for treating colon cancer [35]. Higashi et al. developed a successful cancer vaccine strategy using combined IL18 and HSV-TK suicide gene therapy [36]. However, there is no specific study for the treatment of colon cancer with the suicide gene of thymidine kinase. MSCs expressing thymidine kinase suicide gene has been used for treating glioblastoma [28,30], breast [20,37], and melanoma [38]. Apart from other studies, the main advantage of the present study is monitoring the therapeutic gene expression which was controlled by inducible Tet On system. Suicide gene based therapy can induce side effects to the normal tissues, however, if the suicide gene is delivered only to the targeted cancer cells, it does not impose any harm onto healthy cells. Targeting of cancer by using MSCs as the delivery agent and the inducible system which can regulate the transgene expression, are able to decrease toxicities of the therapy to normal tissues. This inducible system might be useful for treating other cancer types by using suicide gene or other therapeutic genes such as TRAIL, interferons, etc.

The prodrug’s bystander effect is thought to be mediated by the number of MSCs that come into contact with tumor cells, and this may therefore be an important factor in the therapeutic outcome of suicide gene-based therapies using MSCs [39]. In the current study, we injected all suicide gene-transduced MSCs intratumorally, which guaranteed that all tumors received the MSCs. To validate the viability of MSCs after delivery to the tumors, all mice were imaged using BLI to measure Fluc activity for MSC-Tet-TK (1 mg/kg body weight DOX) and without the DOX system MSC-TK (approximately 1 × 10^6^) were injected intra-tumorally after 24 h of DOX induction (or without induction). After treatment with GCV for 5 days and daily DOX injection to ensure stable transgene activation in in the MSC-Tet-TK cells (but not the MSC-TK cells), all of the mice were imaged using BLI again to monitor Fluc activity in order to determine whether the MSCs responded to the GCV (Figure S6A,B). To measure the effect of GCV on the viability of suicide gene-transduced MSCs, a second dose of suicide gene-transduced MSCs was injected on day 6. For the group of animals treated with MSCs having the Tet On system (MSC-Tet-TK) these were treated with DOX and GCV and compared to a group of animals also treated with MSC-Tet-TKs but only treated with DOX. Similarly, for the animals treated with MSCs not having the DOX system (MSC-TK) these were treated with GCV and compared to animals treated with MSC-TKs only. 

Tumor growth in vivo during treatment with MSC-Tet-TK cells was measured using BLI to assess the Rluc activity of the CT26/Rluc cells on days 0, 6, and 13. Rluc activity was found to be decreased at days 6 and 13 in the MSC-Tet-TK+DOX+GCV treatment group relative to the MSC-Tet-TK+DOX (i.e., without GCV) treatment group (Figure 4A). The therapeutic efficiency of MSC-TK cells in this same colon cancer model was also studied. We also observed that Rluc activity was decreased on days 6 and 13 in the MSC-TK+GCV treatment group compared to the MSC-TK only treatment group (Figure 4B). Based on the data, we have successfully created MSCs with a Tet-On system (MSC-Tet-TK) and confirmed their therapeutic effect after induction with DOX in a colon cancer xenograft model. Because colon cancer cells (CT26/Rluc) are known to form fast growing tumors; we elected to sacrifice the mice at day 13, after the second injection of cells and subsequent DOX and GCV treatments. After sacrifice, the tumors were excised, weighed, and the tumor tissue processed for a TUNEL assay. In MSC-Tet-TK treated animals, tumor weights decreased significantly in the DOX(+) GCV treatment group compared to the DOX group alone. Similarly, in MSC-TK treated animals there was a significant reduction in tumor weight relative to the MSC-TK group (Figure 4C). Leng et al. have shown similar data, namely that human umbilical cord-derived MSCs (hUC-MSCs), engineered to express HSV1-TK and injected into tumor-bearing mice, enhanced the therapeutic effect of GCV treatment. Administration of these hUC-MSCs suppressed the angiogenesis pathway, thereby inhibiting the migration and proliferation of MDA-MB-231 cells [20]. In addition, Amano et al. have reported an in vivo experiment where intracranial C6 tumors in Sprague-Dawley rats were injected intratumorally with MSC-TK cells and through the bystander effect produced significant tumor growth suppression and prolonged survival time following GCV administration [40].

Apoptosis is widely thought to be the mechanism through which most cancer therapies induce tumor cell death. The TUNEL assay has been used extensively to assess apoptosis in vivo [41]. In the current study, we used TUNEL staining and found an increased number of apoptotic cells in the tumors from mice treated with MSC-Tet-TK cells +DOX+GCV compared to the tumors from mice treated with MSC-Tet-TK cells +DOX (Figure 5A). Similarly, tumors from mice treated with MSC-TK cells +GCV showed an increase in apoptotic cells compared to tumors from mice treated with MSC-TK cells only (Figure 5B). These results confirm that, in mice bearing a xenograft colon cancer tumor injected with MSCs expressing the HSV1-sr39TK suicide gene (either inducible by DOX or expressed constitutively), GCV enhances apoptotic cell death in the tumor xenograft. Ryu et al. similarly studied the antitumor activity of MSCs-TK treated with valproic acid and found that this activity enhanced apoptotic cell death in intracranial gliomas since the tissue sections from TUNEL-stained glioma-bearing mice showed a significant increase in the number of apoptotic cells [41].

In conclusion, we have successfully produced a Tet-On-inducible therapeutic gene system linked with an optical reporter gene system, which allowed non-invasive monitoring of therapeutic gene expression both in vitro and in vivo. With this innovative reporter gene coupled Tet-On system, we can control expression of the therapeutic gene and minimize the potential for adverse impacts related to gene-based therapies. Experimental studies using various therapeutic genes such as interferon-β [42,43], interleukin-23 [44,45], interleukin-2 [46], cytosine deaminase [35,47], and TNF-related apoptosis-inducing ligand [48] have been reported. Tet system-based noninvasive molecular imaging, a reproducible and controllable imaging tool, could also be used with other therapeutic genes. In order for MSC-based gene therapies to be introduced into the clinic, suicide gene-expressing MSCs must be examined for viability and their inability to promote tumors in vivo. The present study, however, supports the usefulness of MSCs with an inducible suicide gene for the treatment of colon cancer.

## 4. Materials and Methods

### 4.1. Chemicals

pRetroX-TRE3G and pRetroX-Tet3G plasmids, tetracycline-free fetal bovine serum (FBS), and DOX were purchased from Clontech (Mountain View, CA, USA). Gentamicin, a CaPO_4_ transfection kit, and mouse bone marrow-derived MSCs were purchased from Invitrogen (Carlsbad, CA, USA). MSCs were cultured in Dulbecco’s modified Eagle’s medium (DMEM)-F12 (HyClone, Logan, UT, USA) supplemented with 10% FBS (Hyclone), 1× GlutaMAX (Invitrogen), and 1% gentamicin (Gibco-BRL Life Technologies, Gaithersburg, MD, USA). The mouse colon cancer cell line CT26 (American Tissue Culture Collection (Manassas, VA, USA) was grown in DMEM medium supplemented with 10% FBS and 1% penicillin/streptomycin solution (HyClone).

### 4.2. Retroviral Transduction of MSCs

The therapeutic suicide gene (HSV1-sr39TK; a mutant of HSV1-TK) and eGFP (enhanced green fluorescent protein) were linked to create a fusion protein. Firefly luciferase (Fluc2) was inserted after the internal ribosome entry site (IRES) sequence in the pIRES vector and downstream of the HSV1-sr39TK-eGFP fusion protein to create the vector pIRES-HSV1-sr39TK-eGFP-IRES-Fluc2. From this plasmid, the HSV1-sr39TK-eGFP-IRES-Fluc2 sequence was removed and it was inserted after the pTRE3G region into the pRetroX-TRE3G response plasmid. This plasmid encodes a modified Tet-On advanced transactivator protein called Tet-On 3G, which has markedly increased sensitivity to DOX. The inducible promoter pTRE3G consists of seven repeats of a 19-bp Tet operator sequence that results in very low basal expression and high maximal expression of the transduced protein after induction. DOX can activate Tet-On 3G and bind specifically to pTRE3G to promote transcription. The retrovirus in pRetroX-TRE-HSV1-sr39TK-eGFP-IRES-Fluc2 and pRetro-Tet-On 3G was isolated from Gryphon E cells (Allele Biotechnology, San Diego, CA, USA) after separately transfecting the cells using the CaPO_4_ method. Two days after an overnight transfection and a medium change, the medium was collected, filtered through a 0.45 µm filter, and then concentrated using an Amicon Ultra 15 centrifugal filter (Merck Millipore, Burlington, MA, USA). MSCs transduced with a 1:1 dilution of Tet-On3G and Retro-HSV1-sr39TK-eGFP-IRES-Fluc2 were used to make Tet-On MSCs (MSC-Tet-TK/Fluc2 or MSC-Tet-TK) or MSCs without a Tet system (MSC-TK/Fluc2 or MSC-TK). MSC-Tet-TK and MSC-TK cells were sorted based on expression of eGFP. MSC-Tet-TK cells were treated with DOX at 2 µg/mL for 24 h and then sorted. The sorted cells were screened by optical imaging and confocal microscopy.

### 4.3. Lentiviral Transduction of Colon Cancer Cells (CT26)

CT26 cells were transduced with lentiviral particles expressing mCherry-Rluc (Renilla luciferase) under the control of the cytomegalovirus (CMV) promoter (Genecopoeia, Rockville, MD, USA). Positive mCherry cells were selected, and stable clones were selected using Fluorescence activated cell sorting (FACS) Aria III (BD Biosciences, San Jose, CA, USA). The stable cells, named CT26/Rluc, were screened by optical imaging and fluorescence microscopy.

### 4.4. Optical Imaging

Bioluminescent imaging (BLI) was performed using an IVIS Lumina II Imaging System (Perkin Elmer, Waltham, MA, USA). Fluc2 and Rluc activity and the response to therapy was monitored in transfected cells using d-Luciferin and coelenterazine [22] as substrates, respectively. CT26/Rluc and the Fluc activity of MSC-Tet-TK and MSC-TK, respectively, were used to monitor tumor development, with d-Luciferin (150 mg/kg) injected intraperitoneally into mice and after 5 min to evaluate Fluc2 expression and coelenterazine (3 mg/kg) injected intravenously into mice to assess Rluc expression. After injection of coelenterazine, mice were imaged immediately with the IVIS Lumina II Imaging System.

### 4.5. ^3^H-Penciclovir (PCV) Uptake Assay

To confirm HSV1-sr39TK functional activity, we performed a ^3^H-penciclovir uptake assays as described in Sekar et al. 2012 [37]. The transduced MSCs, MSC-TK, and MSC-Tet-TK cells, along with naive MSCs were separately plated (2 × 10^5^ cells/well) in 6-well plates, and after 24 h, cells were induced with or without DOX for a further 24 h and then incubated with 1 μCi of ^3^H-penciclovir (Moravek Biochemicals, La Brea, CA, USA) for 1, 2, or 4 h at 37 °C in an atmosphere containing 5% CO_2_. The cells were then washed twice with ice-cold PBS, lysed in 0.1 mL of 0.1% sodium dodecyl sulfate (SDS), and analyzed in a scintillation counter with 10 mL of scintillation fluid. Activity was measured as counts per minute (CPM) and analyzed based on the CPM of cell lysates/CPM of medium/µg of protein.

### 4.6. CCK8 Assay of Cell Viability

To determine the effect of DOX and GCV on naive cell viability, we performed a CCK8 assay. MSCs, (5 × 10^3^ cells) were seeded into 96-well plates. After incubation overnight, the cells were treated with various doses of DOX and GCV for 48 h. After 48 h, CCK-8 (Dojindo, Kumamoto, Japan) was applied for 1 h, and the developed color was read at 450 nm.

### 4.7. Bystander Effect

To assess the therapeutic efficiency of the suicide gene-transduced MSC-mediated bystander effect, MSC-Tet-TK and MSC-TK cells were separately mixed at a 1:1 ratio with CT26/Rluc cells in a 48-well plate and then induced with or without DOX for 24 h. After 24 h, various concentrations of GCV were applied for a further 48 h. The fate of cells was monitored by BLI of Rluc and Fluc, using IVIS imaging.

### 4.8. Tumor Model

Six-week-old female BALB/c white mice purchased from Central Lab. Animal Inc., Seoul, Korea and mice were housed under standard laboratory conditions. All experimental procedures were reviewed and approved by the Kyungpook National University (KNU-2012-43, KNU-2016-0095, 24 June 2016) Animal Care and Use Committee and performed in accordance with the Guiding Principles for the Care and Use of Laboratory Animals. The mice were injected in the right flank with 1 × 10^6^ CT26/Rluc cells in PBS. One week later, Rluc activity was measured, and mice were randomly separated into groups. For MSC-Tet-TK, the following groups were compared: (1) the MSC-Tet-TK+DOX group and (2) the MSC-Tet-TK+DOX+GCV group. For MSC-TK, the groups were (1) the MSC-TK group and (2) MSC-TK+GCV group. Mice in both groups were injected intra-tumorally with 1 × 10^6^ transduced MSCs (MSC-Tet-TK and MSC-TK), and the day the experiment began was considered day 0. A schematic of the experimental plan is shown in Figure S5. Twenty-four hours after injection of the transduced MSCs, the treatment group mice were intraperitoneally injected with GCV (30 mg/kg/day) on days 1–5. A second dose of MSCs was injected on Day 6, and cells were treated with DOX and GCV on days 7–12. At the end of experiment (day 13), mice were sacrificed and tumors were excised, weighed, fixed with 10% buffered formalin (Sigma, St. Louis, MO 63103, USA), and processed for a TUNEL (Terminal deoxynucleotidyl transferase (TdT) dUTP Nick-End Labeling) assay. Tumor development was evaluated using the BLI of Rluc on days 0, 6, and 13. The transduced MSCs were tracked by BLI of Fluc on days 1 and 5. 

### 4.9. TUNEL Staining for Ex Vivo Tumor

To confirm apoptosis in mice in the GCV-treated group, we performed an in vivo apoptosis assay using an ApopTag Peroxidase In Situ Apoptosis Detection Kit (Millipore, Burlington, MA, USA). This assay identifies apoptotic cells in situ by labeling and detecting DNA strand breaks by the TUNEL method. The results are visualized using bright-field microscopy.

### 4.10. Statistical Analysis

For this study, the data are expressed as mean ± standard deviation (SD). Data from the experimental groups were analyzed by *t*-test using GraphPad Prism 5 software version 5.01 (GraphPad Software, Inc., La Jolla, CA, USA). A P-value of less than 0.05 was considered statistically significant.

## 5. Conclusions

The results of the current study support the use of MSCs with an inducible suicide gene system for the treatment of colon cancer. This system may open new avenues for the use of other inducible suicide gene systems with different levels of bystander effects in preclinical trials.

## Supplementary Materials

The following are available online at https://www.mdpi.com/1422-0067/19/4/1002/s1.
