# Correction: Kalimuthu et al. Regulated Mesenchymal Stem Cells Mediated Colon Cancer Therapy Assessed by Reporter Gene Based Optical Imaging. *Int. J. Mol. Sci.* 2018, *19*, 1002

**DOI:** 10.3390/ijms26178225

**Published:** 2025-08-25

**Authors:** Senthilkumar Kalimuthu, Liya Zhu, Ji Min Oh, Ho Won Lee, Prakash Gangadaran, Ramya Lakshmi Rajendran, Se Hwan Baek, Yong Hyun Jeon, Shin Young Jeong, Sang-Woo Lee, Jaetae Lee, Byeong-Cheol Ahn

**Affiliations:** Department of Nuclear Medicine, School of Medicine, Kyungpook National University, Kyungpook National University Hospital, Daegu 41944, Republic of Korea; senthilbhus@gmail.com (S.K.); liyazhu1001@gmail.com (L.Z.); ojm0366@naver.com (J.M.O.); howonlee1234@gmail.com (H.W.L.); prakashg@knu.ac.kr (P.G.); ramyasep9@gmail.com (R.L.R.); bsh4498@naver.com (S.H.B.); jeon9014@gmail.com (Y.H.J.); syjeongnm@gmail.com (S.Y.J.); swleenm@knu.ac.kr (S.-W.L.); jaetae@knu.ac.kr (J.L.)

The authors wish to make the following correction to this paper [1]. The authors state that the scientific conclusions remain unaffected. This correction was approved by the Academic Editor. The original publication has also been updated.

**Error in Figures**

In the original publication, there was a mistake in Figure 1B,C, and Figure 2 (DOX(−) MSC-Tet-TK) as published. The error occurred due to erroneous copying of images during figure assembly. The corrected version of Figure 1B,C, and Figure 2 (DOX(−) MSC-Tet-TK) appears below.

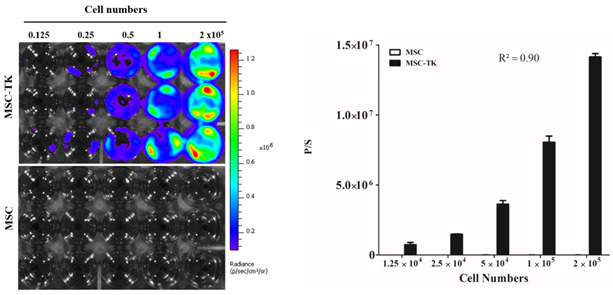

**Figure 1B**

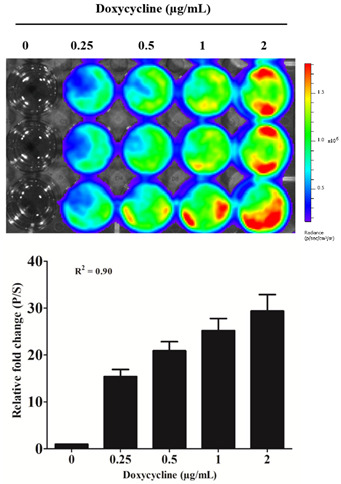

**Figure 1C**

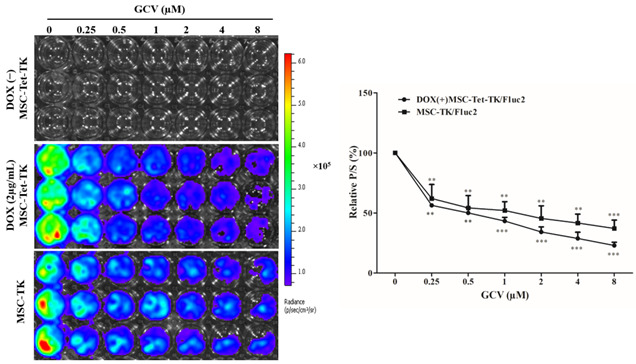

**Figure 2.** (DOX(−) MSC-Tet-TK).

**Error in Figure Legends**

In the original publication, there were typographical errors in the legends for Figures 2 and 3. The correct legends appear below.

**Figure 2.** Fluc activity of MSC-Tet-TK and MSC-TK cells after ganciclovir (GCV) treatment for 48 h. Fluc activity was measured by bioluminescent imaging (BLI), and the quantitation for MSC-Tet-TK and MSC-TK cells is shown in the right-hand panel. Values obtained from three experiments are expressed as the mean ± standard deviation (SD), ** *p* < 0.01, *** *p* < 0.001 (by Student’s *t*-test). p/s, photons/second.

**Figure 3.** Bystander effect of MSC-Tet-TK and MSC-TK cells. (**A**) Rluc activity in co-cultures (1:1) of naive MSCs and CT26/Rluc cells treated with the indicated concentrations of GCV for 48 h. (**B**) BLI images of the Rluc activity and quantitation data of CT26/Rluc in co-cultures (1:1) of MSC-TK or MSC-Tet-TK cells in the absence or presence of doxycycline (DOX(−) and DOX 2 μg/mL, respectively). Three experiments are expressed as the mean ± standard deviation (SD), * *p* < 0.05, ** *p* < 0.01, *** *p* < 0.001 (by Student’s *t*-test). p/s, photons/second.

**Text Correction**

There was an error in the original publication [1]: an error in the figure results. A correction has been made to the Results Section, in the subsection “Characterization of MSC-Tet-TK and MSC-TK”, Paragraph Number 1:

*2.1. Characterization of MSC-Tet-TK and MSC-TK*

The transduced MSC-Tet-TK and MSC-TK cells were created by retroviral transfection, and eGFP-positive cells were sorted by FACS Aria III. Cells with an eGFP concentration of more than 50% were used for further study. The expression of MSC-TK cell eGFP and MSC-Tet-TK cell eGFP after induction with DOX (2 µg/mL) for 24 h (Figure 1A) was confirmed by confocal microscopy images. The morphology of MSC-Tet-TK cells are heterogeneous and not uniform, because MSCs are a heterogeneous cell population. MSC-Tet-TK cells with eGFP positive revealed more rod-like morphology than others, which might be related to the genetic alteration. Some of the MSC-TK cells revealed higher eGFP signals due to higher eGFP expression, because protein synthesis can vary with individual cells, withdifferent copy numbers of the transduced gene. The Fluc activity of MSC-TK cells increased with increasing cell numbers (Figure 1B, *R*^2^ = 0.90). The BLI signal in MSC-Tet-TK cells increased 15-, 21-, 25-, and 29-fold with increasing concentrations of DOX (0.25, 0.5, 1, and 2 µg/mL), whereas no signal was observed in DOX(−) cells (Figure 1C). Furthermore, we found that Fluc activity increased 17-, 19-, 20-, 20-, 15-, and 7-fold with increasing DOX treatment (0.5 to 4 µg/mL) for 48 h and then decreased at 8 and 16 µg/mL DOX (Figure S1). Therefore, in this study, we elected to use 2 µg/mL DOX for transgene activation.
